# Clinical and pathophysiological roles of lower lobe–dominant mucus plugs on computed tomography in patients with asthma with and without bronchiectasis

**DOI:** 10.1016/j.jacig.2025.100566

**Published:** 2025-09-04

**Authors:** Naoya Tanabe, Hisako Matsumoto, Natsuko Nomura, Yusuke Hayashi, Ryo Sakamoto, Mikio Toyoshima, Osamu Matsuno, Toshiyuki Kita, Nobuyuki Hizawa, Takuro Sakagami, Koichi Fukunaga, Mari Miki, Naozumi Hashimoto, Noboru Hattori, Sumito Inoue, Kazuto Matsunaga, Kojiro Otsuka, Takahiro Tsuburai, Hiroaki Iijima, Hiroyuki Nagase, Morio Nakamura, Tetsuji Kawamura, Takashi Kijima, Taisuke Tsuji, Hironobu Sunadome, Susumu Sato, Atsuyasu Sato, Toyohiro Hirai

**Affiliations:** a Department of Respiratory Medicine, Kyoto University Graduate School of Medicine, Kyoto, Japan; b Department of Respiratory Medicine & Allergology, Kindai University Faculty of Medicine, Osakasayama, Japan; c Department of Respiratory Medicine, Takatsuki Red Cross Hospital, Takatsuki, Japan; d Department of Diagnostic Imaging and Nuclear Medicine, Graduate School of Medicine, Kyoto University, Kyoto, Japan; e Department of Respiratory Medicine, Hamamatsu Rosai Hospital, Hamamatsu, Japan; f Department of Allergy and Rheumatoid Disease, Osaka Habikino Medical Center, Osaka, Japan; g Department of Respiratory Medicine, NHO Kanazawa Medical Center, Kanazawa, Japan; h Department of Pulmonary Medicine, Institute of Medicine, University of Tsukuba, Tsukuba, Japan; i Department of Respiratory Medicine, Faculty of Life Sciences, Kumamoto University, Kumamoto, Japan; j Division of Pulmonary Medicine, Department of Medicine, Keio University School of Medicine, Tokyo, Japan; k Department of Respiratory Medicine, NHO Toneyama Medical Center, Osaka, Japan; l Department of Internal Medicine, Tokushima Prefecture Naruto Hospital, Tokushima, Japan; m Department of Respiratory Medicine, Nagoya University Graduate School of Medicine, Nagoya, Japan; n Department of Respiratory Medicine, Fujita Health University, Toyoake, Aichi, Japan; o Department of Molecular and Internal Medicine, Graduate School of Biomedical and Health Sciences, Hiroshima University, Hiroshima, Japan; p Department of Cardiology, Pulmonology, and Nephrology, Yamagata University Faculty of Medicine, Yamagata, Japan; q Department of Respiratory Medicine and Infectious Disease, Graduate School of Medicine, Yamaguchi University, Yamaguchi, Japan; r Department of Respiratory Medicine, Shinko Hospital, Kobe, Japan; s Department of Respiratory Medicine, St Marianna University Yokohama Seibu Hospital, Yokohama, Japan; t Department of Respiratory Medicine, St Marianna University School of Medicine, Kawasaki, Japan; u Department of Respiratory Medicine, Tsukuba Medical Center Hospital, Tsukuba, Japan; v Division of Respiratory Medicine and Allergology, Department of Medicine, Teikyo University School of Medicine, Tokyo, Japan; w Department of Pulmonary Medicine, Tokyo Saiseikai Central Hospital, Tokyo, Japan; x Department of Respiratory Medicine, NHO Himeji Medical Center, Himeji, Japan; y Department of Respiratory Medicine and Hematology, Hyogo Medical University, Hyogo, Japan; z Department of Respiratory Medicine, Japanese Red Cross Kyoto Daiichi Hospital, Kyoto, Japan; aa Department of Respiratory Care and Sleep Control Medicine, Graduate School of Medicine, Kyoto University, Kyoto, Japan

**Keywords:** Asthma, comorbid bronchiectasis, exacerbations, exhaled nitric oxide, lower lobe dominance, lung function, mucus plug, spatial distribution

## Abstract

**Background:**

Despite the clinical relevance of mucus plugging, the role of spatial distribution of mucus plugs remains unclear in patients with asthma.

**Objective:**

We sought to examine whether greater lower lobe mucus plug dominance is associated with more clinical and pathophysiological impairments in 2 cohorts, including patients with and without bronchiectasis.

**Methods:**

Patients with asthma without and with clinical diagnosis of bronchiectasis underwent chest computed tomography at Kyoto University Hospital (Kyoto cohort) and Japanese multicenters (bronchiectasis and asthma [BEXAS] cohort), respectively. Mucus plugs in airways were visually scored on computed tomography, and the difference in mucus plug score between lower and upper-middle lobes (Δ mucus plug score) was calculated.

**Results:**

Among 176 (Kyoto) and 42 (BEXAS) enrolled patients, 82 and 33 exhibited mucus plug scores greater than or equal to 1, respectively. Higher Δ mucus plug score was associated with lower percentage of the predicted FEV_1_ and the presence of exacerbation history in both cohorts. Higher Δ mucus plug score was associated with luminal narrowing of the fifth-generation, but not the third- or fourth-generation, lower lobe airways in the Kyoto cohort, and bronchiolitis score in the BEXAS cohort. In the multivariable model, higher Δ mucus plug score was associated with symptoms and exacerbations, independent of whole-lung mucus plug score in the Kyoto cohort.

**Conclusions:**

Lower lobe–dominant mucus plugs were associated with lower lung function and exacerbations in patients with asthma, irrespective of comorbid bronchiectasis. The spatial distribution of mucus plugs additionally to whole-lung mucus plug score may help to understand clinical roles of mucus plugging in asthma.

Asthma is a chronic airway disease, characterized by airway inflammation and variable clinical presentations.^1^ Despite advancements in medical interventions, including biologics, a substantial number of patients with asthma remain symptomatic, experiencing exacerbations and having impaired lung function.^2^, ^3^, ^4^ This highlights the need for a deeper understanding of the factors associated with low lung function and exacerbations. Airway mucus plugging was first described in autopsy findings of fatal asthma and subsequently quantified on chest computed tomography (CT).^5^ Mucus plugs in the airways impair lung function, increase the risk of exacerbation, and persist in the same bronchial segments over time, serving as an important biomarker of asthma.^6^, ^7^, ^8^, ^9^, ^10^

Emerging evidence suggests that the spatial distribution of mucus plugs is worth addressing in the context of asthma pathophysiology. On ultrahigh-resolution computed tomography (U-HRCT), which allows more accurate measurements of wall thickness of airways peripheral to subsegmental airways than conventional HRCT,^11^ mucus plugging is associated with wall thickening of these airways, but not segmental and subsegmental airways.^12^ Moreover, mucus plugs are more frequently found in the lower lobes than in the upper lobes,^9^ and specific lung segments with mucus plugging exhibit greater wall thickening and ventilation defects than those without mucus plugging.^13^^,^^14^ Because local ventilation may be more impaired in the lower lungs than in the upper lungs as asthma severity increases,^15^ it was hypothesized that lower lobe–dominant mucus plugging could be associated with more severe pathophysiological changes and exacerbations in patients with asthma. However, little is known about the clinical roles of upper-lower lobe distributions of mucus plugging in patients with asthma.

In addition to the mechanical impacts related to the distinct spatial distribution of mucus plugs, their association with airway inflammatory types should be clarified for effective asthma management. Mucus plugging is typically associated with eosinophils and type 2 inflammation in asthma^16^ and chronic obstructive airway diseases.^17^^,^^18^ However, a more complex inflammatory process is proposed in approximately 20% of mucus plugging cases.^16^ Indeed, in patients with refractory asthma with comorbid bronchiectasis, the association between the whole-lung mucus plug score and type 2 inflammation was influenced by the presence of neutrophils and monocytes, making it less straightforward.^19^ Further analysis of the spatial distribution of mucus plugs is needed to understand their association with inflammatory types in asthma.

Taken together, we aimed to examine whether a difference in mucus plug score between lower and upper/middle lobes (Δ mucus plug score) could be associated with lower lung function, the presence of exacerbations, exhaled nitric oxide (Feno), and other clinical and pathophysiological features by using 2 cohorts, including a cohort of patients with asthma without a clinical diagnosis of bronchiectasis and another cohort of patients with asthma with bronchiectasis.

## Methods

### Study design and population

This study cross-sectionally analyzed data sets from 2 cohorts including (1) an ongoing single-center cohort that prospectively enrolled patients with asthma without clinical diagnosis of bronchiectasis and underwent U-HRCT scans during exacerbation-free stable period in an outpatient clinic of Kyoto University Hospital in Japan between 2023 and 2024 (Kyoto cohort) and (2) the BEXAS cohort, a nationwide survey that retrospectively enrolled patients with refractory asthma with bronchiectasis between 2015 and 2019.^19^^,^^20^ Details of inclusion and exclusion criteria of the bronchiectasis and asthma (BEXAS) cohort are provided in this article’s Online Repository at www.jaci-global.org. In this study, to exclude allergic bronchopulmonary aspergillosis/mycosis (ABPA/ABPM), patients who had results of *Aspergillus* sensitization tests and had HRCT images suitable for mucus score evaluation were included. In the Kyoto cohort, patients with asthma, diagnosed according to the Global INitiative for Asthma guideline,^1^ underwent U-HRCT, postbronchodilator spirometry, self-reported questionnaires including Asthma Control Test (ACT)^21^ and modified Medical Research Council (mMRC), and measurements of Feno, white blood cell differentials, and blood IgE. In both cohorts, patients with concurrent respiratory disease other than chronic obstructive pulmonary disease, a history of lung resection, ABPA/ABPM based on Asano’s clinical diagnostic criteria,^22^ insufficient CT quality such as poor breath-holding during scans, or incidental abnormal CT findings such as interstitial lung abnormalities were excluded. This study was conducted in accordance with the Declaration of Helsinki and approved by the Ethics Committee (nos. R2911-2 and R2168). Written informed consent was obtained from all participants.

### Clinical and spirometry data

In the Kyoto cohort, body mass index, smoking history, asthma duration, and exacerbation frequency in the past year were confirmed at enrollment. Exacerbation requiring systemic corticosteroids was defined as symptomatic worsening requiring additional use of systemic corticosteroids for at least 3 days.^23^ Postbronchodilator spirometry was performed using the CHESTAC-8900 (Chest MI Corp, Tokyo, Japan) following the American Thoracic Society/European Respiratory Society guidelines^24^ and normalized using the Japanese predicted values of forced vital capacity and FEV_1_.^25^ Feno was measured using the NIOX VERO (Circassia Pharmaceuticals, Oxford, United Kingdom) following the American Thoracic Society/European Respiratory Society recommendation.^26^ In the BEXAS cohort, data on demographic characteristics, spirometry, Feno, blood, and the number of exacerbations requiring antibiotics or corticosteroids in the past 2 years were collected from the attending physicians, as previously described.^19^

### CT scan acquisition

In the Kyoto cohort, full-inspiratory U-HRCT images at 1024 × 1024 matrix with 0.25-mm slice thickness were obtained using an Aquilion Precision scanner (Canon Medical, Tokyo, Japan) with 120 kVp and autoexposure control and then reconstructed using the Advanced Intelligent Clear-IQ Engine (Canon Medical, Tokyo, Japan) .^27^ In the BEXAS cohort, images at 512 × 512 matrix with 0.5- to 1.25-mm slice thickness were reconstructed using sharp kernels to visually evaluate mucus plugs and bronchiolitis.

### CT image analyses

Airway mucus plugs were identified and scored without knowledge of clinical information by visual inspection by 2 pulmonary physicians according to a scoring system based on bronchopulmonary segment anatomy.^6^ The mucus plug scores obtained by the 2 inspectors were averaged for the whole lung, upper-middle lobes (right upper and middle lobes and left upper lobe), and lower lobes (right and left lower lobes). Details are presented in the Online Repository. The Δ mucus plug score was calculated by subtracting the score of the upper-middle lobes from the score of the lower lobes. The range of Δ mucus plug scores was from −9 to +9. A higher Δ mucus plug score indicated more dominant mucus plugs in the lower lobes.

In the BEXAS cohort, chest CT data obtained during the exacerbation-free stable period as well as during exacerbations were included. Mucus plugs were assessed across all bronchopulmonary segments, including those with bronchiectasis. Bronchiolitis and degree of bronchodilatation were also scored. Bronchiolitis was defined as the presence of centrilobular nodules or tree-in-bud signs in 1 or more lobes, and its severity was assessed by counting the number of affected lobes, ranging from 0 to 5. The degree of bronchodilatation was assessed using the modified Reiff score.^28^^,^^29^

In the Kyoto cohort, lumen area (LA) and wall area (WA) were measured at all identifiable branches of the third (segmental), fourth (subsegmental), and fifth (sub-subsegmental) generations of the right apical (RB1) and posterior basal (RB10) paths. The WA% was calculated as the percentage of WA to the sum of WA and LA.^12^^,^^30^, ^31^ LA was normalized by body surface area (BSA).

### Statistical analysis

Continuous variables are expressed as mean ± SD or median (interquartile range). Categorical variables are expressed as absolute numbers and percentages. Normality of the dependent variables in the models was visually confirmed using histograms. Continuous and categorical variables were compared between 2 groups using *t* test or the Mann-Whitney test. Correlations were tested using Spearman correlation coefficient (rs). Multivariable logistic regression and Poisson regression models were constructed to examine whether Δ mucus plug score was associated with the presence of symptoms defined as an ACT score less than 20 or 23, the presence of dyspnea defined as an mMRC score less than 1 or 2, the presence of exacerbations, and the number of exacerbations in the past year. Statistical analysis was performed using R software version 4.2.3 (R Foundation for Statistical Computing, Vienna, Austria). Statistical significance was set at a *P* value less than .05.

## Results

**Fig 1:**
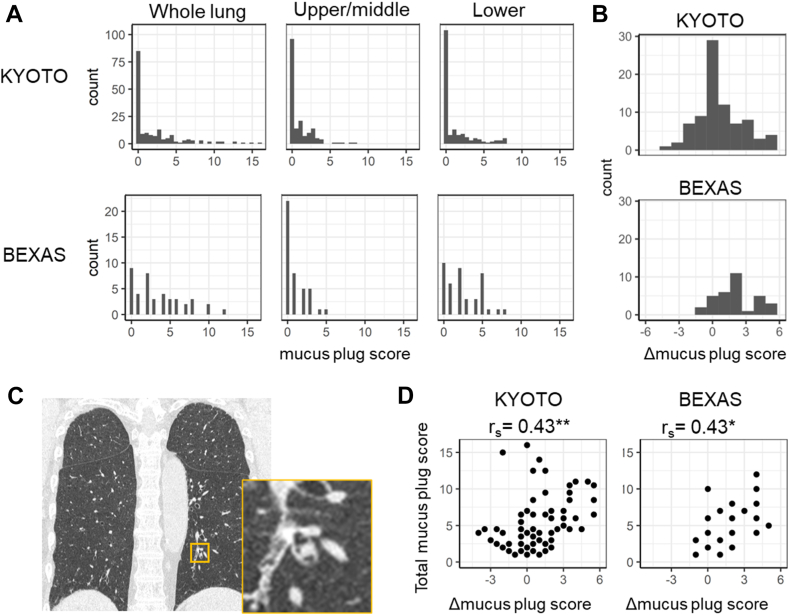
**A-D,** Histograms of mucus plug score (Fig 1, *A*), lower-upper differences in the score (Δ mucus plug score; Fig 1, *B*), representative CT with lower lobe–dominant mucus plugs (Fig 1, *C*), and associations between Δ mucus plug score and whole-lung mucus plug score (total mucus plug score; Fig 1, *D*) in the Kyoto and BEXAS cohorts.

Of the 197 patients initially enrolled, 22 were excluded because of ABPA/ABPM, active bacterial pneumonia, history of lung lobectomy, and poor quality CT images, and 176 with asthma without a clinical diagnosis of bronchiectasis were included in the Kyoto cohort. Of the 67 patients in the BEXAS cohort who had *Aspergillus* sensitization test and CT image results, 25 were excluded because the slice thickness (≥2.0 mm) of the CT images was inappropriate for accurate mucus plug evaluation, and 42 with asthma with bronchiectasis but not ABPA/ABPM were included in this study. Among them, 82 and 33 patients in the Kyoto and BEXAS cohorts, respectively, had at least 1 mucus plug on CT and thus were included in subsequent analyses. Their characteristics are provided in Table I. As shown in Fig 1, the rate of mucus plug presence was greater in the BEXAS group than in the Kyoto cohort (78.6% vs 46.6%; *P* = .0002). For patients with mucus plugs, the Δ mucus plug score was greater in the BEXAS cohort than in the Kyoto cohort (median, 2.00 vs 0.50; *P* = .001). The mucus plug score in the whole lung (total mucus plug score) was significantly correlated with the Δ mucus plug score in both the Kyoto and BEXAS cohorts (rs = 0.43 and 0.43, respectively). In the BEXAS study, timings of CT scans were available for 28 patients with mucus plugs: 17 underwent scans during the stable period and 11 during exacerbations. Because total mucus plug, Δ mucus plug, and bronchiolitis scores were comparable between the stable and exacerbation phases (see Table E1 in this article’s Online Repository at www.jaci-global.org), data obtained during both phases were analyzed together. Separate analyses for the stable and exacerbation periods are presented in Table E2 (in the Online Repository available at www.jaci-global.org).

**Table I:** Characteristics of all patients and patients with mucus plugs in 2 cohorts

Characteristics	Overall		Total mucus plug score (≥1)	
	Kyoto (asthma) (N = 176)	BEXAS (asthma + bronchiectasis) (N = 42)	Kyoto (N = 82)	BEXAS (N = 33)
Age (y)	65.6 ± 13.4	68.8 ± 15.5	70.9 ± 10.1	68.2 ± 15.8
Male, n (%)	74 (42)	17 (41)	35 (43)	11 (33)
Percentage of the predicted FEV_1_ (%)	90.2 ± 20.3	94.4 ± 29.3	83.6 ± 18.1	92.4 ± 25.5
BMI (kg/m^2^)	24.9 ± 4.1	22.3 ± 4.4	23.9 ± 3.8	21.6 ± 3.9
N/F/C smoker, n (%)	94/68/14 (53/39/8)	31/11/0 (74/26/0)	40/36/6 (49/44/7)	27/6/0 (82/18/0)
Smoking pack year	10.7 ± 16.8	3.8 ± 10.7	12.4 ± 18.2	3.8 ± 11.5
Asthma-onset age (y)	45.3 ± 19.1	34.9 ± 26.0	48.1 ± 17.9	36.0 ± 25.9
Feno (ppb)	23.0 (13.5-39.0)	22.0 (18.0-44.0)	36.0 (19.00-60.00)	22.0 (16.0-46.5)
BEC (/μL)	150.5 (60.5-315.8)	149.9 (71.3-342.5)	229.8 (101.3-532.0)	224.9 (81.6-456.3)
IgE (IU/mL)	150.0 (38.0-540.0)	183.0 (93.3-506.5)	300.0 (99.00-800.0)	147.3 (62.5-519.0)
BEC ≥ 500 (/μL), n (%)	28 (16)	8 (19)	24 (29)	7 (21)
Total IgE ≥ 417 IU/mL, n (%)	53 (30)	11 (26)	34 (42)	7 (21)
Asp-IgE, n (%)	21 (12)	21 (50)	13 (16)	16 (49)
Reiff score	0.0 (0.0-2.0)	3.5 (0.0-6.0)	0.0 (0.0-1.5)	3.0 (0.0-6.0)
MP score	0.5 (0.0-3.1)	2.5 (1.0-5.8)	3.5 (2.0-6.4)	4.0 (2.0-6.0)
MP upper lobes	0.0 (0.0-0.5)	0.0 (0.0-0.0)	0.5 (0.0-1.4)	0.0 (0.0-1.0)
MP upper-middle lobes	0.0 (0.0-1.5)	0.0 (0.0-2.0)	1.5 (1.0-2.5)	1.0 (0.0-2.0)
MP lower lobes	0.0 (0.0-1.5)	2.0 (1.0-4.8)	2.0 (1.0-4.0)	3.0 (2.0-5.0)
Biologics, n (%)	72 (41)	11 (26)	28 (34)	7 (21)
IL-5/IL-4α/IgE/TSLP, n (%)	20/24/23/5 (11/14/13/3)	8/3/0/0 (20//7/0/0)	10/11/7/0 (12/13/9/0)	4/3/0/0 (12/9/0/0)
ICS, n (%)	172 (98)	36 (90)	80 (98)	27 (87)
LAMA, n (%)	95 (54)	22 (52)	43 (52)	18 (55)
LABA, n (%)	144 (82)	32 (76)	64 (78)	24 (73)
OCS, n (%)	13 ( 7)	11 (27)	7 (9)	8 (24)
Macrolide, n (%)	31 (18)	23 (55)	15 (18)	20 (61)

Values are expressed as mean ± SD or median (interquartile range) unless otherwise indicated. *Asp-IgE*, Positivity of anti-IgE for *Aspergillus*; *BEC*, blood eosinophil count; *BMI*, body mass index; *ICS*, inhaled corticosteroid; *IL-5*, mepolizumab and benralizumab that block IL-5 or IL-5 receptor; *IL-4α*, dupilumab that blocks the IL-4α receptor; *LABA*, long-acting β-agonist; *LAMA*, long-acting muscarinic antagonist; *MP*, mucus plug; *N/F/C*, never/former/current (smoker); *OCS*, oral corticosteroid; *TSLP*, tezepelumab that blocks the thymic stromal lymphopoietin pathway.

**Fig 2:**
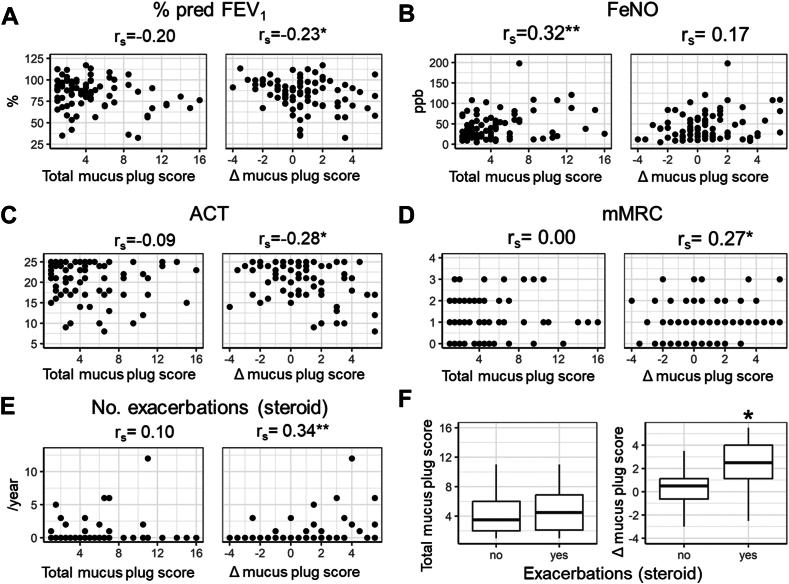
**A-F,** Associations of total mucus plug score and score difference between lower lobes and upper-middle lobes (Δ mucus plug score) with percentage of the predicted FEV_1_ (Fig 2, *A*), Feno (Fig 2, *B*), ACT score (Fig 2, *C*), mMRC score (Fig 2, *D*), and number (Fig 2, *E*) and presence (Fig 2, *F*) of exacerbations requiring additional systemic steroid in patients with asthma (Kyoto). ∗*P* < .05; ∗∗*P* < .01.

As shown in Fig 2, patients with mucus plugs in the Kyoto cohort showed that higher Δ mucus plug score was significantly associated with a lower percentage of the predicted FEV_1_ (rs = −0.23). Total mucus plug score was significantly associated with Feno (rs = 0.32), but not with the percentage predicted FEV_1_. Higher Δ mucus plug score, but not total mucus plug score, was significantly associated with lower ACT score (rs = −0.28), higher mMRC score (rs = 0.27), and greater number of exacerbations requiring additional systemic steroids in the past year (rs = 0.34). Moreover, Δ mucus plug score, but not total mucus plug score, was higher in those with a history of exacerbations than in those without. In multivariable models (Table II), higher Δ mucus plug score was significantly associated with higher odds ratios of ACT scores less than 20 or 23, mMRC scores of 1 or higher, the presence of exacerbation history, and greater number of exacerbations independent of total mucus plug score. These associations remained significant after adjustment for age, sex, and smoking pack year.

**Table II:** Multivariable models for associations of total mucus plug score and Δ mucus plug score with clinical parameters in patients with mucus plugs (n = 82; Kyoto)

Models	Total mucus plug score	Δ mucus plug score
Logistic models	Odds ratio (95% CI)	Odds ratio (95% CI)
ACT score < 20 (basic)	0.98 (0.83 to 1.13)	1.41 (1.08 to 1.89)∗
ACT score < 20 (basic + demographics)	1.06 (0.88 to 1.27)	1.36 (1.04 to 1.85)∗
ACT score < 23 (basic)	0.93 (0.81 to 1.07)	1.41 (1.10 to 1.87)†
ACT score < 23 (basic + demographics)	0.94 (0.81 to 1.09)	1.41 (1.09 to 1.87)∗
mMRC ≥ 1 (basic)	0.96 (0.82 to 1.11)	1.46 (1.11 to 1.99)∗
mMRC ≥ 1 (basic + demographics)	1.03 (0.88 to 1.23)	1.46 (1.09 to 2.04)∗
mMRC ≥ 2 (basic)	0.87 (0.70 to 1.03)	1.21 (0.96 to 1.73)
mMRC ≥ 2 (basic + demographics)	0.97 (0.76 to 1.21)	1.26 (0.92 to 1.76)
Exacerbations ≥ 1 (basic)	0.84 (0.62 to 1.06)	1.93 (1.31 to 3.20)†
Exacerbations ≥ 1 (basic + demographics)	0.88 (0.63 to 1.14)	1.81 (1.22 to 3.00)†

**Poisson model**	**Estimate (95% CI)**	**Estimate (95% CI)**

No. of exacerbations/past year (basic)	−0.01 (−0.13 to 0.11)	0.48 (0.32 to 0.64)†
No. of exacerbations/past year (basic + demographics)	0.03 (−0.09 to 0.14)	0.39 (0.23 to 0.57)†

Multivariable linear regression, logistic, or Poisson models were constructed. Basic models included using mucus plug score in the entire lung and lower-upper lobe difference in mucus plug score (Δ mucus plug score) as independent variables. Demographic factors, including age, sex, and smoking pack years, were added as independent variables to these models, referred to as “Basic + demographics.”

∗ *P* < .05.

† *P* < .01.

Regarding the associations with airway dimensions as provided in Table III, higher Δ mucus plug score was significantly associated with higher WA% and lower LA/BSA of the fifth generation of the RB10 path. These associations were not found in the third and fourth generations of the RB10 path and the third, fourth, and fifth generations of the RB1 path.

**Table III:** Associations of mucus plug score and regional difference with airway dimensions in patients with asthma (Kyoto)

Aiway dimension	Total mucus plug score∗	Δ mucus plug score
RB1 path†		
WA% (3rd)	0.11	0.21
WA% (4th)	−0.08	0.04
WA% (5th)	−0.01	0.11
LA/BSA (3rd)	0.03	−0.18
LA/BSA (4th)	0.05	−0.16
LA/BSA (5th)	0.04	−0.17
RB10 path†		
WA% (3rd)	0.12	0.08
WA% (4th)	0.17	0.19
WA% (5th)	0.14	0.25‡
LA/BSA (3rd)	0.02	−0.11
LA/BSA (4th)	−0.04	−0.22
LA/BSA (5th)	−0.04	−0.25‡

*RB1*, Right apical path; *RB10*, right lower posterior path.

∗ Whole-lung mucus plug score.

† Airways: 3rd, segmental airways; 4th, subsegmental airways; 5th, sub-subsegmental airways.

‡ *P* < .05.

**Fig 3:**
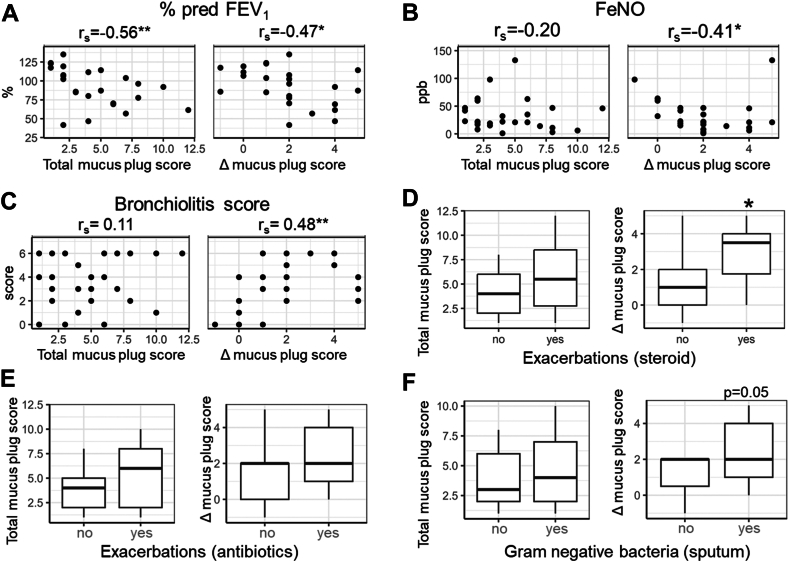
Associations of total mucus plug score and score difference between lower lobes and upper-middle lobes (Δ mucus plug score) with percentage of the predicted FEV_1_ (Fig 3, *A*), Feno (Fig 3, *B*), bronchiolitis score (Fig 3, *C*), and presence of exacerbations requiring additional systemic steroid (Fig 3e, *D*), antibiotics (Fig 3, *E*), and gram-negative bacteria (Fig 3, *F*) detected in sputum in patients with asthma with bronchiectasis (BEXAS). ∗*P* < .05; ∗∗*P* < .01.

Finally, in patients with asthma with bronchiectasis in the BEXAS cohort (as shown in Fig 3), higher Δ mucus plug score, in addition to higher total mucus plug score, was significantly associated with lower percentage of the predicted FEV_1_ (rs = −0.47 and −0.56, respectively). The Δ mucus plug score, but not total mucus plug score, was associated negatively with Feno and positively with bronchiolitis score (rs = 0.41 and 0.48, respectively) as shown in Fig 3, *B* and *C*, but not with the modified Reiff score (rs = −0.14; see Fig E1 in this article’s Online Repository at www.jaci-global.org). Furthermore, Δ mucus plug score, but not total mucus plug score, was significantly greater in patients with a history of exacerbations requiring systemic steroid than those without. The Δ mucus plug score did not differ between patients with and without a history of exacerbations requiring antibiotics. The number of exacerbations requiring antibiotics in the past 2 years (n = 28) was associated with bronchiolitis score (rs = 0.51) and Feno (rs = −0.53). The Δ mucus plug score was insignificantly greater in patients with positive sputum culture of gram-negative bacteria than those without (*P* = .05). When the analysis was confined to cases with CT scans obtained during the stable phase, the correlation between Δ mucus plug score and the number of exacerbations requiring systemic steroid remained significant and was close to significance with the number of exacerbations requiring antibiotics (Table E2). In addition, the association between bronchiolitis score and the number of exacerbations requiring antibiotics was significant (*ρ* = 0.54; *P* = .04).

## Discussion

This study used 2 cohorts and consistently demonstrated that higher Δ mucus plug score (ie, lower lobe–dominant mucus plug score) was associated with lower percentage of the predicted FEV_1_ and the presence of a history of exacerbations requiring systemic steroids in patients with asthma irrespective of comorbid bronchiectasis. Moreover, higher Δ mucus plug score was associated with greater WA% and lower LA/BSA in the fifth, but not third or fourth, generation of lower lobe airways in the Kyoto cohort and bronchiolitis score in the BEXAS cohort. To our knowledge, this is the first study to examine the association of greater lower lobe dominance of mucus plugging with lower lung function, exacerbations, and relatively peripheral airway disease in patients with asthma. Furthermore, the association between Δ mucus plug score and Feno differed between the 2 cohorts, with a negative association observed in the BEXAS cohort. These findings indicate that understanding the spatial distribution of mucus plugging and its underlying inflammation is crucial for the management of asthma with or without bronchiectasis.

The total mucus plug score and Δ mucus plug score were greater in patients in the BEXAS group than in those in the Kyoto cohort, suggesting that the coexistence of bronchiectasis could facilitate mucus plugs, especially in the lower lobe airways, in asthma. Nonetheless, lower lobe–dominant mucus plugs were consistently associated with lower percentage of the predicted FEV_1_ and the history of exacerbations across both cohorts. In addition, in the Kyoto cohort, symptoms assessed as ACT, dyspnea assessed as mMRC, and exacerbations were significantly associated with higher Δ mucus plug score, even after adjusting for total mucus plug score. In a single-photon emission CT study using technetium-99m labeled ultrafine carbon particles, patients with asthma exhibited a lower lobe–dominant patchy distribution of regions affected by airway closure, which may have been induced by airway inflammation, mucus plugging, and increased smooth muscle tone.^32^ Moreover, ventilation was greater in the lower lobes than in the upper lobes.^33^ It is possible that luminal occlusion by mucus plugging, particularly in the lower lobes, may decrease lower lobe ventilation, increase the extent of patchy airway closures, impair lung function, and enhance a risk of exacerbation, which further imposes symptomatic burdens in patients with asthma.

Higher WA% and lower LA/BSA in the fifth-generation airways of the RB10 path, but not in the RB1 path or the third- and fourth-generation airways, were associated with Δ mucus plug score, but not total mucus plug score, in the Kyoto cohort. The accurate measurement of the lumen and wall sizes of the fifth-generation airways is an advantage of the use of U-HRCT in this study because conventional CT potentially causes the underestimation of the lumen size and the overestimation of the wall size of the relatively peripheral airways.^34^ In a previous conventional CT study, the presence of mucus plugs in the left lower lobe path, but not in other paths, was associated with a lower LA of the third-generation airways in the corresponding path of 33 patients with asthma or chronic obstructive pulmonary disease.^13^ Moreover, mucus plugs in airways, particularly in the seventh-generation airways or more proximal generation airways, are associated with lower FEV_1_, and the removal of mucus plugs by biologics increases FEV_1_ in patients with asthma.^6^^,^^9^^,^^10^^,^^35^ In the BEXAS cohort, we found the association of higher Δ mucus plug score with greater bronchiolitis score. This is consistent with a previous report showing that small airway disease may be critical of the pathogenesis of bronchiectasis.^36^ In subanalysis by CT timings, the statistical significance of the association between Δ mucus plug and bronchiolitis scores on stable-phase CT disappeared because of the small sample size, but the strength of the correlation was not substantially affected (*ρ* = 0.37; *P* = .14). Collectively, the present findings suggest that lower lobe–dominant mucus plugging may be associated with luminal narrowing and wall thickening of relatively peripheral lower lobe airways in patients with asthma.

Besides the importance of spatial distribution, understanding the inflammatory milieu behind mucus formation is crucial for better management of asthma, given its heterogeneous nature. Higher Feno was associated with higher total mucus plug score, but not Δ mucus plug score, in the Kyoto cohort. The positive association between Feno and total mucus plug score in patients with asthma alone is consistent with previous reports on this association and the concept that Feno reflects the level of IL-13 that induces mucin production from airway epithelial cells,^6^^,^^8^^,^^12^ but this association may not be restricted to the lower lobes.

In contrast, Feno was negatively associated with Δ mucus plug score in the BEXAS cohort, particularly with Δ mucus plug score obtained during the exacerbation period (Table E2), extending previous findings that lower Feno is associated with bronchiectasis alone^37^ or as a comorbid of severe asthma with productive cough.^38^ Various mechanisms may account for the lower Feno: nitric oxide (NO) fails to diffuse across the increased and viscous secretions in the airways or NO is consumed through reactions with reactive oxygen species^37^ and by denitrifying organisms, including *Pseudomonas*. In patients with cystic fibrosis, NO synthase expression is decreased and asymmetric dimethyl arginine level, which inhibits NO synthase activity, is increased in the lower airways.^39^ The same might occur in patients with bronchiectasis and those with asthma complicated by bronchiectasis, although further analysis is required. Moreover, lower Feno was associated with a history of exacerbations requiring antibiotics, and greater Δ mucus plug score was associated with sputum gram-negative bacteria positivity and greater CT bronchiolitis score. Therefore, the association between Δ mucus plug score and lower Feno suggests that lower lobe–dominant mucus plugs may reflect the presence of chronic bacterial infection and/or colonization in asthma with bronchiectasis. A recent report on the association between sputum microbiome and mucus plugs in stable patients with chronic obstructive airway diseases showed that an increase in the relative abundance of genus *Haemophilus* was associated with mucus plugging in the milieu of moderate eosinophilic inflammation.^17^ We believe that although regulating type 2 inflammation is essential, clinicians should also pay attention to avoid overuse of corticosteroid and to control bacterial colonization in the airways in management of mucus plugs in patients with asthma and comorbid bronchiectasis.

This study had some limitations. First, the sample sizes of the cohorts, particularly the BEXAS cohort, were relatively small. However, the associations of Δ mucus plug score with lower FEV_1_ and exacerbations were detected in the 2 cohorts, suggesting the validity of the results. Second, because of inconsistent CT scanning conditions in the BEXAS cohort, we could not perform quantitative measurement of airway dimensions. Nonetheless, the associations of bronchiolitis score with Δ mucus plug score in the BEXAS cohort are consistent with the associations of WA% and LA/BSA in the fifth, but not third or fourth, generations with Δ mucus plug score in the Kyoto cohort. We believe that the CT findings in the BEXAS cohort are important because of the currently limited data on asthma with bronchiectasis, despite their clinical impact. Because of the significantly different clinical characteristics between the Kyoto and BEXAS cohorts, it is essential to reassess the findings from each cohort using a validation cohort that includes patients with more closely aligned clinical characteristics. Third, whether patients with primary ciliary dyskinesia were included in the BEXAS cohort is unclear because of lack of genotyping data. Lastly, the BEXAS cohort included cases with CT data obtained during the exacerbation period. However, the correlations between the number of exacerbations requiring antibiotics and Δ mucus plug or bronchiolitis score on stable-phase CT suggest that findings on stable-phase CT reflect chronic inflammation/infection rather than acute infection.

### Conclusion

Greater lower lobe–dominant mucus plugs were associated with lower percentage of the predicted FEV_1_ and exacerbations similarly in patients with asthma with and without bronchiectasis. The different involvement of type 2 inflammation with lower lobe–dominant mucus plugs between asthma with and without bronchiectasis suggest that a more individualized approach to remove mucus plugs should be considered according to the extent of bronchiectasis in patients with asthma. The observed associations of Δ mucus plug score with symptomatic burdens and exacerbations, independent of total mucus plug score, suggest the clinical relevance of CT-based identification of lower lobe–dominant mucus plugging for better management of the disease.

### Declaration of AI and AI-assisted technologies in the writing process

During the preparation of this work, the authors used DeepL Pro to improve readability and language of the work. After using this service, the authors reviewed and edited the content as needed and take full responsibility for the content of the publication.

## Disclosure statement

This study was partially supported by grants from 10.13039/501100002424Fujifilm Corporation, the 10.13039/501100001691Japan Society for the Promotion of Science (Grants-in-Aid for Scientific Research; grant no. 22K08233), and the Scientific Assembly of Allergy, Immunology & Inflammation, Japanese Respiratory Society.

Disclosure of potential conflict of interest: N. Tanabe received research grants from Daiichi Sankyo and Fujifilm Co, Ltd, and lecturer fees from Sanofi K.K., AstraZeneca K.K., and GlaxoSmithKline, outside the submitted work. H. Matsumoto received lecturer fees from Sanofi K.K., AstraZeneca K.K., GlaxoSmithKline, Kyorin Pharmaceutical Co, and Boehringer Ingelheim; received grants from Kyorin Pharmaceutical Co, Boehringer Ingelheim, and Teijin Pharma, outside the submitted work; and received support from the Japanese Respiratory Society and Research Grant from Novartis Japan. O. Matsuno received lecturer fees from Sanofi K.K., AstraZeneca K.K., and GlaxoSmithKline. K. Fukunaga received lecturer fees from Sanofi K.K., AstraZeneca K.K., GlaxoSmithKline, Kyorin Pharmaceutical Co, Boehringer Ingelheim, and Novartis Pharma KK, outside the submitted work; and received grants from Boehringer Ingelheim and Chugai Pharmaceutical, outside the submitted work. N. Hashimoto received research grants from the Japan Science and Technology Age and Nippon Boehringer Ingelheim, outside the submitted work. N. Hattori received lecturer fees from Sanofi K.K., AstraZeneca K.K., GlaxoSmithKline, Kyorin Pharmaceutical Co, Ono Pharmaceutical Co, MSD, and Pfizer Japan, outside the submitted work. S. Inoue received research grant from Kyorin Pharmaceutical Co and lecture fees from Novartis Pharma K.K., AstraZeneca K.K., GlaxoSmithKline, Boehringer Ingelheim (Japan), Kyorin Pharmaceutical Co, and Sanofi K.K., outside the submitted work. K. Matsunaga received lecturer fees from Sanofi K.K., AstraZeneca K.K., and Kyorin Pharmaceutical Co, outside the submitted work. K. Otsuka received lecture fees from Boehringer Ingelheim, Kyorin Pharmaceutical Co, and Novartis Pharma K.K., outside the submitted work. H. Iijima received lecture fees from AstraZeneca K.K., Kyorin Pharmaceutical Co, MSD, and Asahi Kasei Pharma Corp, outside the submitted work. H. Nagase received lecturer fees from Sanofi K.K., AstraZeneca K.K., GlaxoSmithKline, Kyorin Pharmaceutical Co, and Novartis Pharma K.K., outside the submitted work; received grants from GlaxoSmithKline; and serves on the advisory board of Sanofi K.K., AstraZeneca K.K., and GlaxoSmithKline. T. Sakagami received lecturer fees from AstraZeneca K.K., GlaxoSmithKline, Novartis Pharma K.K., and Boehringer Ingelheim, outside the submitted work. T. Kijima received lecturer fees from AstraZeneca K.K. and GlaxoSmithKline. T. Hirai received lecturer fees from AstraZeneca K.K., Kyorin Pharmaceutical Co, and Boehringer Ingelheim, outside the submitted work. The rest of the authors declare that they have no relevant conflicts of interest.
