# Supplementary material for: Clinical and pathophysiological roles of lower lobe–dominant mucus plugs on computed tomography in patients with asthma with and without bronchiectasis
Source: J Allergy Clin Immunol Glob. 2025 Sep 4;4(4):100566. doi: 10.1016/j.jacig.2025.100566 (PMC12509975; doi:10.1016/j.jacig.2025.100566)
Supplement: Supplementary Data [file mmc1.docx]

**Supplementary Information**

**Clinical and pathophysiological roles of lower lobe-dominant mucus plugs on computed tomography in patients with asthma with and without bronchiectasis**

**Supplementary Methods**

**Study design and population**

In the BEXAS cohort, the diagnosis of bronchiectasis as a comorbidity was not based solely on radiological findings but also required persistent sputum symptoms despite standard treatment and management of asthma, irrespective of inhaled corticosteroid doses^1^. Patients were enrolled by attending physicians at the participating institutions contributing to the BEXAS cohort based on their clinical judgment of refractoriness and airway comorbidities. Bronchiectasis was defined as an enlarged bronchoarterial ratio of >1.1 or lack of tapering of an airway toward the periphery. Cases with traction bronchiectasis due to fibrotic interstitial pneumonia, non-tuberculous mycobacterial diseases, cystic fibrosis, and acute bronchiolitis were excluded.

**CT image analyses**

Mucus plug score ranged from 0 to 18 for the whole lung (10 and 8 segments in the right and left lungs, respectively), 0 to 9 for the upper/middle lobes (5 and 4 segments in the right and left lungs), and 0 to 9 for the lower lobes (5 and 4 segments in the right and left lungs). Intraclass correlation coefficients for the two inspectors were 0.73 [0.67, 0.79] and 0.88 [0.84, 0.90] for the mucus plug scores from the upper/middle lobe and lower lobe were, respectively.

The modified Reiff score was assessed according to the presence and extent of airway dilation (tubular = 1, varicose = 2, cystic = 3) across six regions (right upper, middle, lower, left upper, lingula, and lower lobes) and summed (score 0-18). Mucus plug score and Reiff scores obtained in our previous study^1^ were used in the BEXAS cohort of this study.

**Supplementary Figure E1. Associations of total mucus plug score and Δ mucus plug score with Reiff score**


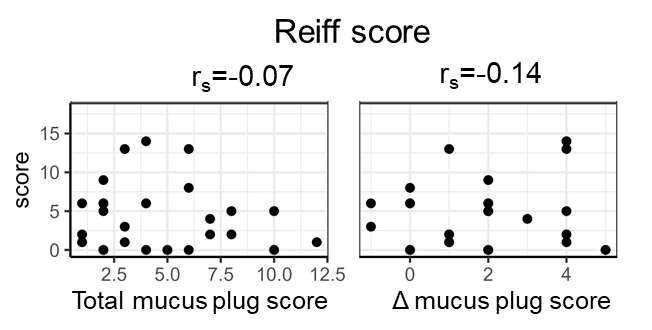


Associations of total mucus plug score and score difference between the lower lobes and upper-middle lobes (Δ mucus plug score ) with the Reiff score on computed tomography were assessed using the Spearman correlation test. Rs = Spearman's correlation coefficient.

**Table E1. CT scores in patients with mucus plugs from the BEXAS cohort, stratified by the timing of CT scans.**

| Scores | Stable phase  N = 17 | Exacerbation period  N = 11 | *P* value |
| --- | --- | --- | --- |
| Total mucus plug | 3 [2, 5] | 4 [1, 6] | 0.87 |
| Δ mucus plug | 2 [0.5, 2] | 2 [1, 4] | 0.68 |
| Bronchiolitis | 3 [2, 5.5] | 4 [3, 6] | 0.27 |

The median [interquartile range] is presented. For five patients, data were unavailable due to transfer of attending physicians.

**Table E2. Associations between Δ mucus plug score and clinical factors** **in patients with mucus plugs from the BEXAS cohort, stratified by the timing of CT scans.**

| Cases with CT scans obtained during the stable period | rs | *P* value |
| --- | --- | --- |
| % of the predicted FEV_1_ | -0.05 | 0.86 |
| FeNO | -0.39 | 0.14 |
| Bronchiolitis score | 0.37 | 0.14 |
| Number of exacerbations requiring systemic steroid | 0.73 | 0.003 |
| Number of exacerbations requiring antibiotics | 0.52 | 0.055 |
|  |  |  |
| Cases with CT scans obtained during the exacerbation period | **rs** | ***P* value** |
| % of the predicted FEV_1_ | -0.73 | 0.06 |
| FeNO | -0.91 | 0.002 |
| Bronchiolitis score | 0.50 | 0.12 |
| Number of exacerbations requiring systemic steroid | 0.11 | 0.77 |
| Number of exacerbations requiring antibiotics | 0.06 | 0.86 |

FeNO = fractional exhaled nitric oxide, FEV_1_ = forced expiratory volume in 1 s.

**Supplementary Reference**

1. Nomura N, Matsumoto H, Asano K, Hayashi Y, Yokoyama A, Nishimura Y, et al. Refractory phenotype of Aspergillus-sensitized asthma with bronchiectasis and allergic bronchopulmonary aspergillosis. J Allergy Clin Immunol Glob 2025; 4:100364.
